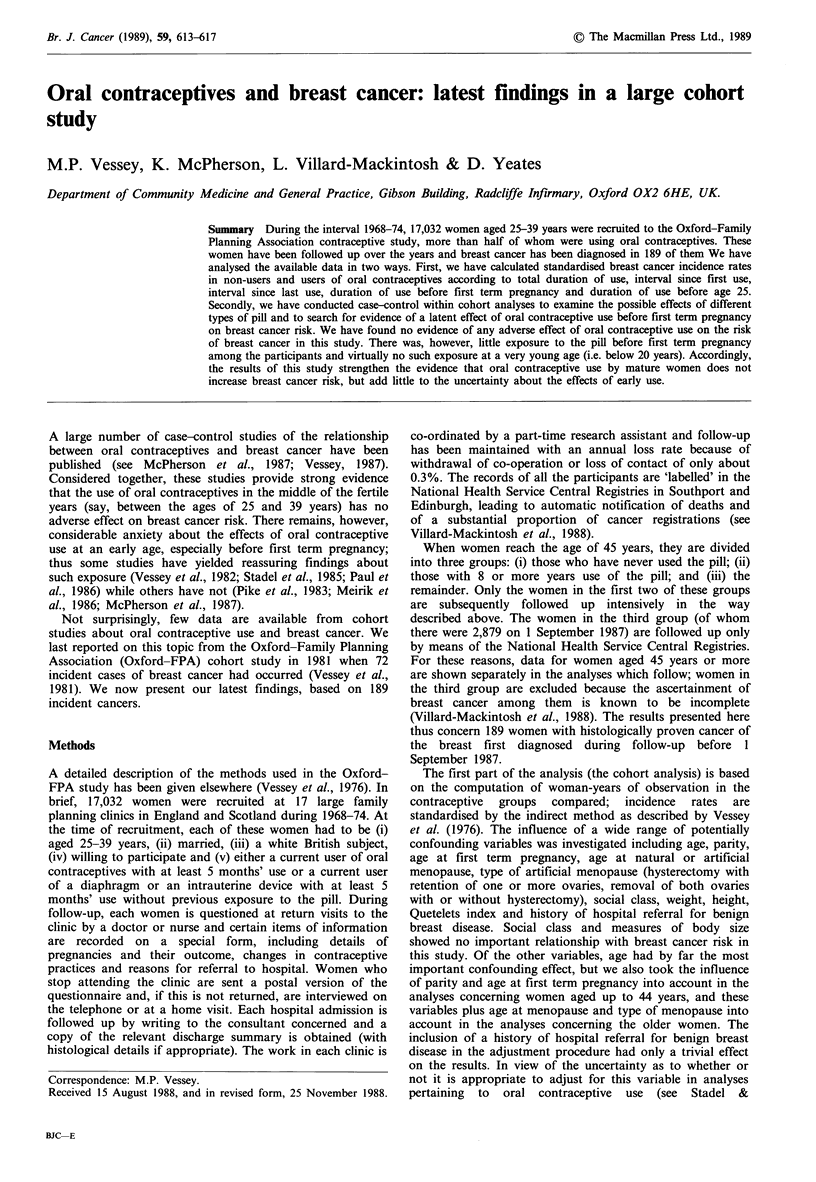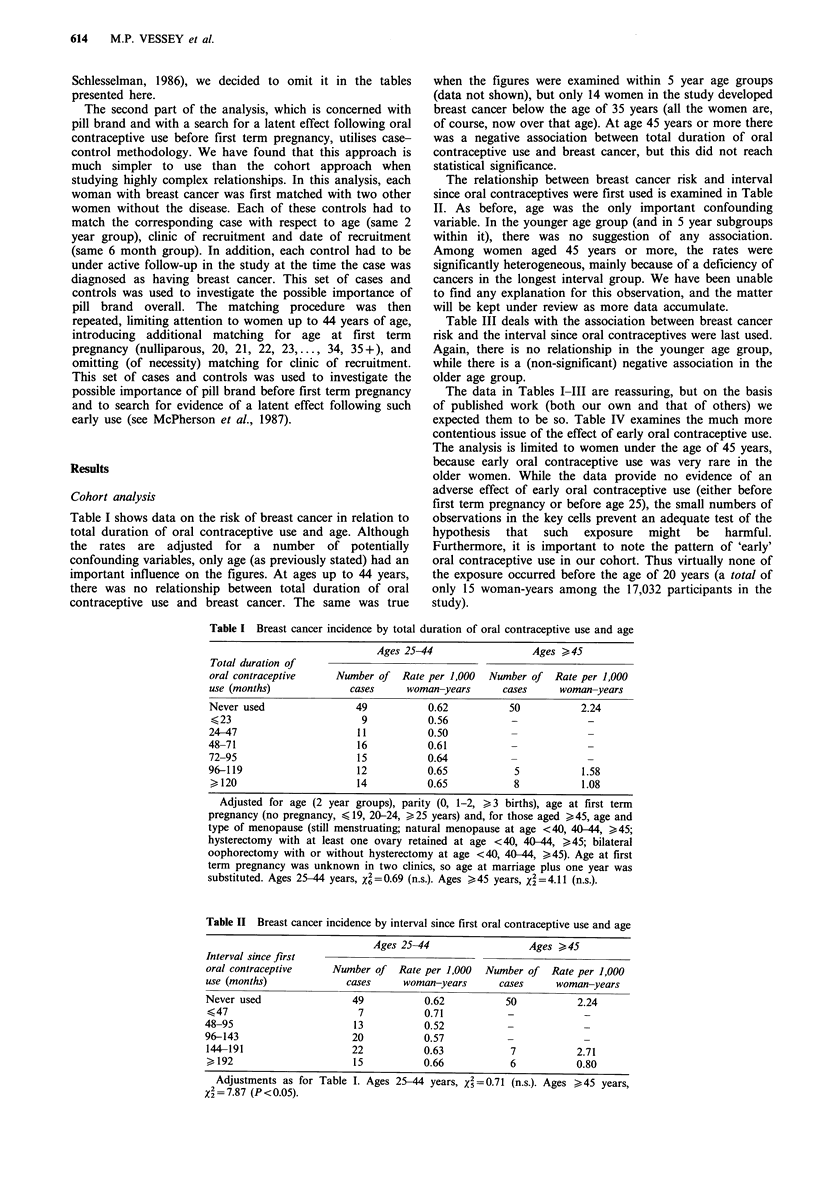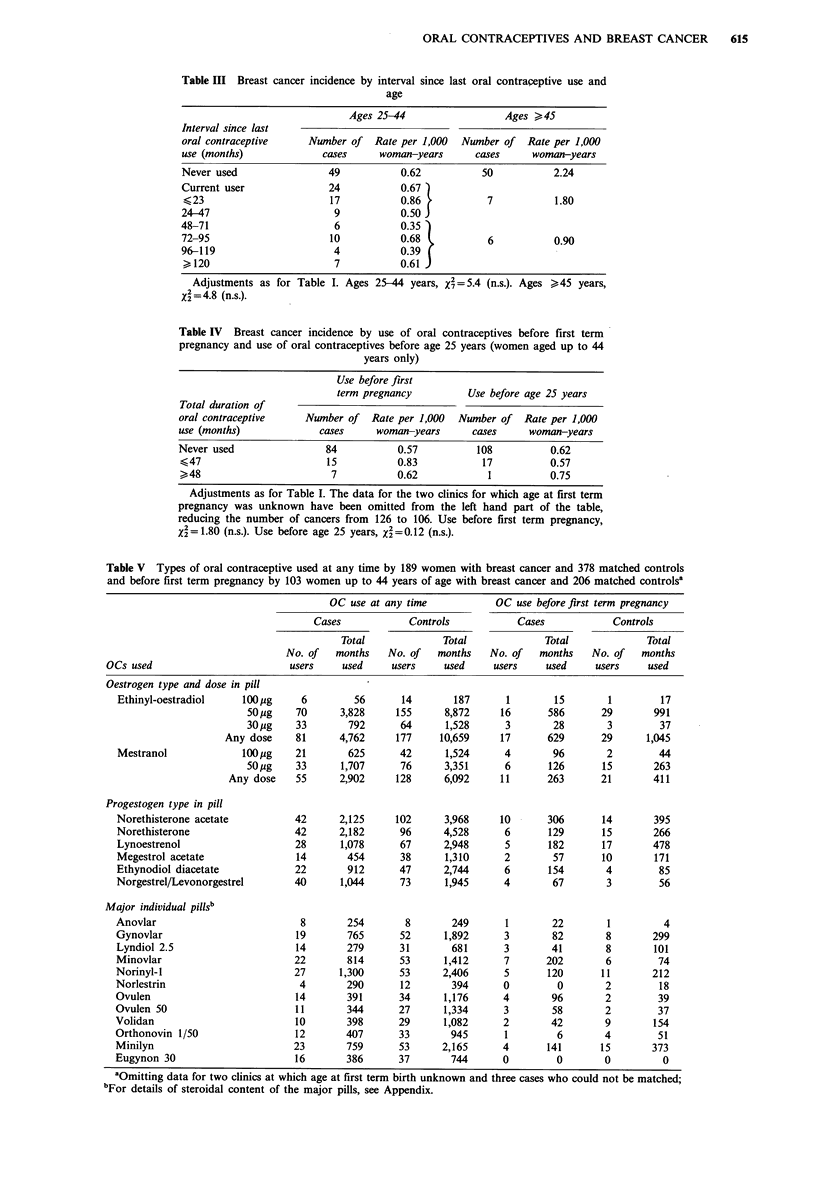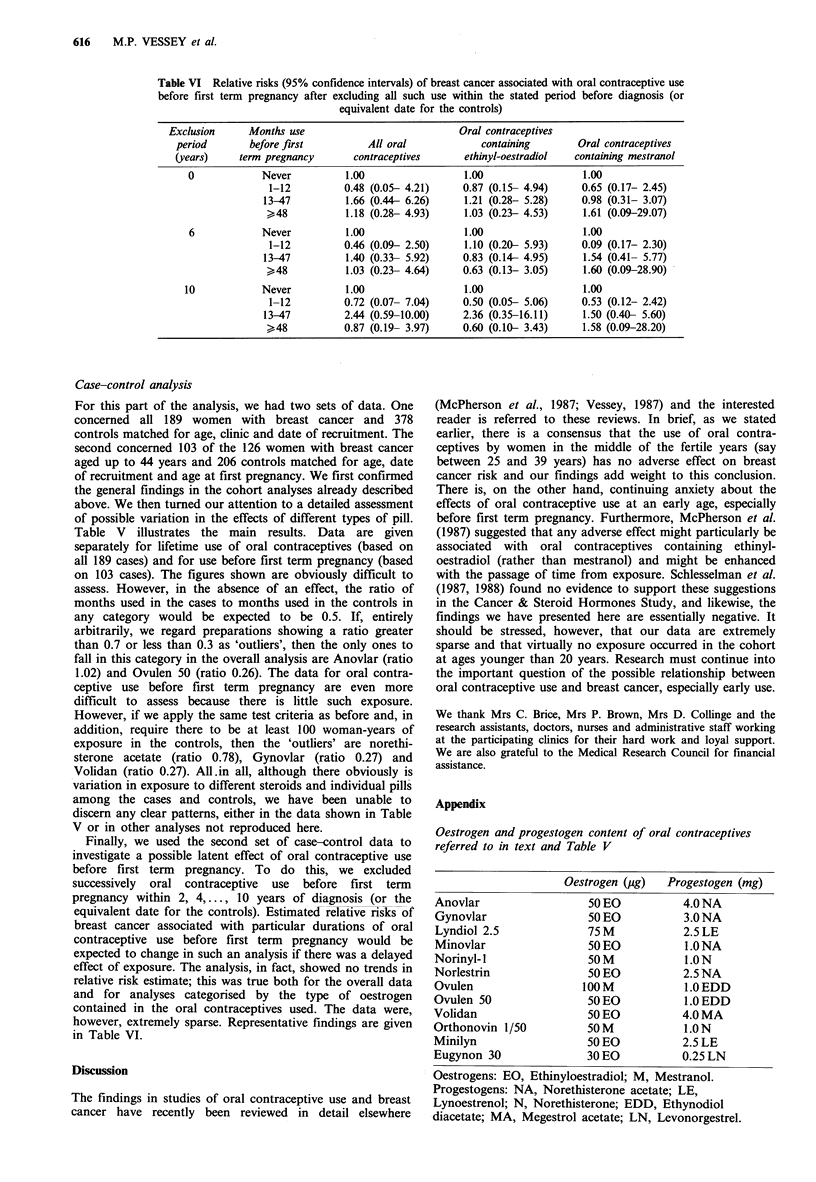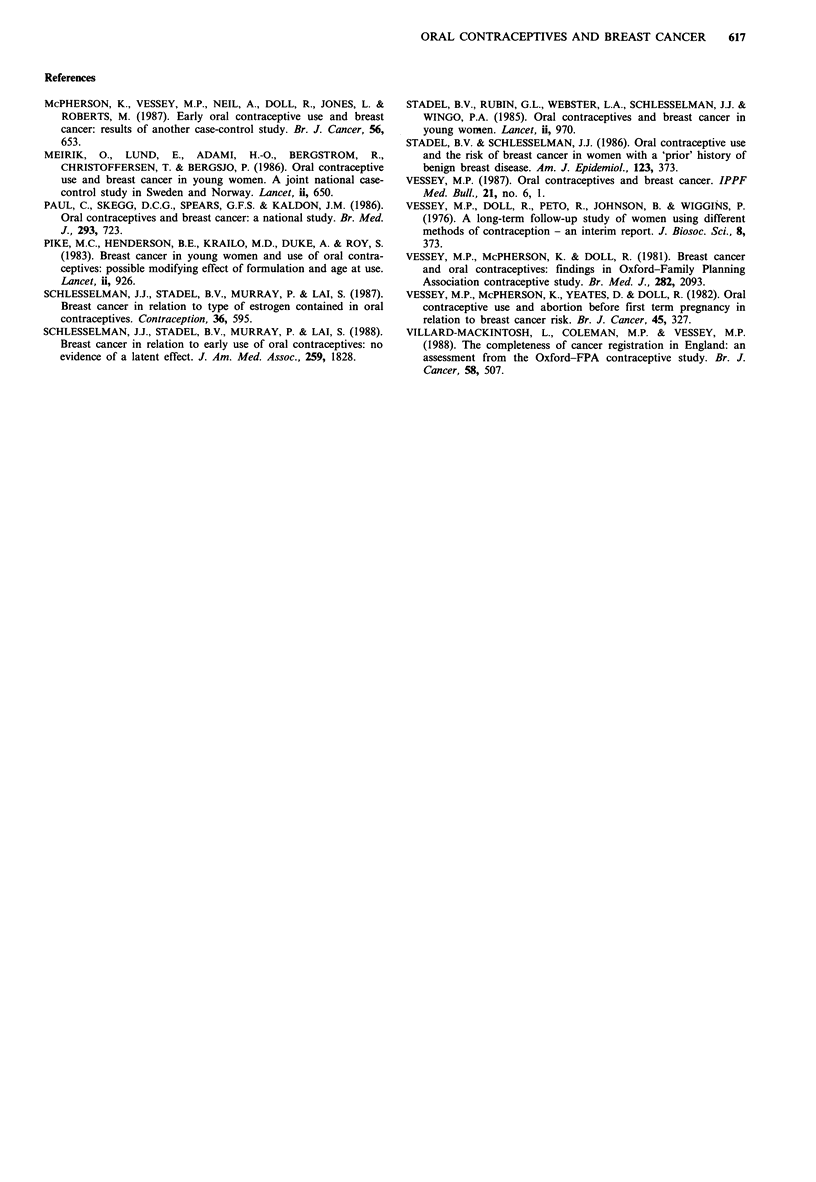# Oral contraceptives and breast cancer: latest findings in a large cohort study.

**DOI:** 10.1038/bjc.1989.124

**Published:** 1989-04

**Authors:** M. P. Vessey, K. McPherson, L. Villard-Mackintosh, D. Yeates

**Affiliations:** Department of Community Medicine and General Practice, Radcliffe Infirmary, Oxford, UK.

## Abstract

During the interval 1968-74, 17,032 women aged 25-39 years were recruited to the Oxford-Family Planning Association contraceptive study, more than half of whom were using oral contraceptives. These women have been followed up over the years and breast cancer has been diagnosed in 189 of them. We have analysed the available data in two ways. First, we have calculated standardised breast cancer incidence rates in non-users and users of oral contraceptives according to total duration of use, interval since first use, interval since last use, duration of use before first term pregnancy and duration of use before age 25. Secondly, we have conducted case-control within cohort analyses to examine the possible effects of different types of pill and to search for evidence of a latent effect of oral contraceptive use before first term pregnancy on breast cancer risk. We have found no evidence of any adverse effect of oral contraceptive use on the risk of breast cancer in this study. There was, however, little exposure to the pill before first term pregnancy among the participants and virtually no such exposure at a very young age (i.e. below 20 years). Accordingly, the results of this study strengthen the evidence that oral contraceptive use by mature women does not increase breast cancer risk, but add little to the uncertainty about the effects of early use.


*Br. J. Cancer* (1989), **59**, 613–617 © The Macmillan Press Ltd., 1989

### Oral contraceptives and breast cancer: latest findings in a large cohort study

M.P. Vessey, K. McPherson, L. Villard-Mackintosh & D. Yeates

*Department of Community Medicine and General Practice, Gibson Building, Radcliffe Infirmary, Oxford OX2 6HE, UK.*

**Summary** During the interval 1968–74, 17,032 women aged 25–39 years were recruited to the Oxford–Family Planning Association contraceptive study, more than half of whom were using oral contraceptives. These women have been followed up over the years and breast cancer has been diagnosed in 189 of them We have analysed the available data in two ways. First, we have calculated standardised breast cancer incidence rates in non-users and users of oral contraceptives according to total duration of use, interval since first use, interval since last use, duration of use before first term pregnancy and duration of use before age 25. Secondly, we have conducted case–control within cohort analyses to examine the possible effects of different types of pill and to search for evidence of a latent effect of oral contraceptive use before first term pregnancy on breast cancer risk. We have found no evidence of any adverse effect of oral contraceptive use on the risk of breast cancer in this study. There was, however, little exposure to the pill before first term pregnancy among the participants and virtually no such exposure at a very young age (i.e. below 20 years). Accordingly, the results of this study strengthen the evidence that oral contraceptive use by mature women does not increase breast cancer risk, but add little to the uncertainty about the effects of early use.

A large number of case–control studies of the relationship between oral contraceptives and breast cancer have been published (see McPherson *et al.*, 1987; Vessey, 1987). Considered together, these studies provide strong evidence that the use of oral contraceptives in the middle of the fertile years (say, between the ages of 25 and 39 years) has no adverse effect on breast cancer risk. There remains, however, considerable anxiety about the effects of oral contraceptive use at an early age, especially before first term pregnancy; thus some studies have yielded reassuring findings about such exposure (Vessey *et al.*, 1982; Stadel *et al.*, 1985; Paul *et al.*, 1986) while others have not (Pike *et al.*, 1983; Meirik *et al.*, 1986; McPherson *et al.*, 1987).

Not surprisingly, few data are available from cohort studies about oral contraceptive use and breast cancer. We last reported on this topic from the Oxford–Family Planning Association (Oxford–FPA) cohort study in 1981 when 72 incident cases of breast cancer had occurred (Vessey *et al.*, 1981). We now present our latest findings, based on 189 incident cancers.

### Methods

A detailed description of the methods used in the Oxford–FPA study has been given elsewhere (Vessey *et al.*, 1976). In brief, 17,032 women were recruited at 17 large family planning clinics in England and Scotland during 1968–74. At the time of recruitment, each of these women had to be (i) aged 25–39 years, (ii) married, (iii) a white British subject, (iv) willing to participate and (v) either a current user of oral contraceptives with at least 5 months' use or a current user of a diaphragm or an intrauterine device with at least 5 months' use without previous exposure to the pill. During follow-up, each women is questioned at return visits to the clinic by a doctor or nurse and certain items of information are recorded on a special form, including details of pregnancies and their outcome, changes in contraceptive practices and reasons for referral to hospital. Women who stop attending the clinic are sent a postal version of the questionnaire and, if this is not returned, are interviewed on the telephone or at a home visit. Each hospital admission is followed up by writing to the consultant concerned and a copy of the relevant discharge summary is obtained (with histological details if appropriate). The work in each clinic is co-ordinated by a part-time research assistant and follow-up has been maintained with an annual loss rate because of withdrawal of co-operation or loss of contact of only about 0.3%. The records of all the participants are 'labelled' in the National Health Service Central Registries in Southport and Edinburgh, leading to automatic notification of deaths and of a substantial proportion of cancer registrations (see Villard-Mackintosh *et al.*, 1988).

When women reach the age of 45 years, they are divided into three groups: (i) those who have never used the pill; (ii) those with 8 or more years use of the pill; and (iii) the remainder. Only the women in the first two of these groups are subsequently followed up intensively in the way described above. The women in the third group (of whom there were 2,879 on 1 September 1987) are followed up only by means of the National Health Service Central Registries. For these reasons, data for women aged 45 years or more are shown separately in the analyses which follow; women in the third group are excluded because the ascertainment of breast cancer among them is known to be incomplete (Villard-Mackintosh *et al.*, 1988). The results presented here thus concern 189 women with histologically proven cancer of the breast first diagnosed during follow-up before 1 September 1987.

The first part of the analysis (the cohort analysis) is based on the computation of woman-years of observation in the contraceptive groups compared; incidence rates are standardised by the indirect method as described by Vessey *et al.* (1976). The influence of a wide range of potentially confounding variables was investigated including age, parity, age at first term pregnancy, age at natural or artificial menopause, type of artificial menopause (hysterectomy with retention of one or more ovaries, removal of both ovaries with or without hysterectomy), social class, weight, height, Quetelets index and history of hospital referral for benign breast disease. Social class and measures of body size showed no important relationship with breast cancer risk in this study. Of the other variables, age had by far the most important confounding effect, but we also took the influence of parity and age at first term pregnancy into account in the analyses concerning women aged up to 44 years, and these variables plus age at menopause and type of menopause into account in the analyses concerning the older women. The inclusion of a history of hospital referral for benign breast disease in the adjustment procedure had only a trivial effect on the results. In view of the uncertainty as to whether or not it is appropriate to adjust for this variable in analyses pertaining to oral contraceptive use (see Stadel &

Correspondence: M.P. Vessey.
Received 15 August 1988, and in revised form, 25 November 1988.

Schlesselman, 1986), we decided to omit it in the tables presented here.

The second part of the analysis, which is concerned with pill brand and with a search for a latent effect following oral contraceptive use before first term pregnancy, utilises case–control methodology. We have found that this approach is much simpler to use than the cohort approach when studying highly complex relationships. In this analysis, each woman with breast cancer was first matched with two other women without the disease. Each of these controls had to match the corresponding case with respect to age (same 2 year group), clinic of recruitment and date of recruitment (same 6 month group). In addition, each control had to be under active follow-up in the study at the time the case was diagnosed as having breast cancer. This set of cases and controls was used to investigate the possible importance of pill brand overall. The matching procedure was then repeated, limiting attention to women up to 44 years of age, introducing additional matching for age at first term pregnancy (nulliparous, 20, 21, 22, 23,..., 34, 35+), and omitting (of necessity) matching for clinic of recruitment. This set of cases and controls was used to investigate the possible importance of pill brand before first term pregnancy and to search for evidence of a latent effect following such early use (see McPherson *et al.*, 1987).

### Results

### *Cohort analysis*

Table I shows data on the risk of breast cancer in relation to total duration of oral contraceptive use and age. Although the rates are adjusted for a number of potentially confounding variables, only age (as previously stated) had an important influence on the figures. At ages up to 44 years, there was no relationship between total duration of oral contraceptive use and breast cancer. The same was true when the figures were examined within 5 year age groups (data not shown), but only 14 women in the study developed breast cancer below the age of 35 years (all the women are, of course, now over that age). At age 45 years or more there was a negative association between total duration of oral contraceptive use and breast cancer, but this did not reach statistical significance.

The relationship between breast cancer risk and interval since oral contraceptives were first used is examined in Table II. As before, age was the only important confounding variable. In the younger age group (and in 5 year subgroups within it), there was no suggestion of any association. Among women aged 45 years or more, the rates were significantly heterogeneous, mainly because of a deficiency of cancers in the longest interval group. We have been unable to find any explanation for this observation, and the matter will be kept under review as more data accumulate.

Table III deals with the association between breast cancer risk and the interval since oral contraceptives were last used. Again, there is no relationship in the younger age group, while there is a (non-significant) negative association in the older age group.

The data in Tables I–III are reassuring, but on the basis of published work (both our own and that of others) we expected them to be so. Table IV examines the much more contentious issue of the effect of early oral contraceptive use. The analysis is limited to women under the age of 45 years, because early oral contraceptive use was very rare in the older women. While the data provide no evidence of an adverse effect of early oral contraceptive use (either before first term pregnancy or before age 25), the small numbers of observations in the key cells prevent an adequate test of the hypothesis that such exposure might be harmful. Furthermore, it is important to note the pattern of 'early' oral contraceptive use in our cohort. Thus virtually none of the exposure occurred before the age of 20 years (a *total* of only 15 woman-years among the 17,032 participants in the study).

**Table I** Breast cancer incidence by total duration of oral contraceptive use and age

| *Total duration of oral contraceptive use (months)* | *Ages 25–44* | | *Ages ≥45* | |
|---|---|---|---|---|
| | *Number of cases* | *Rate per 1,000 woman–years* | *Number of cases* | *Rate per 1,000 woman–years* |
| Never used | 49 | 0.62 | 50 | 2.24 |
| ≤23 | 9 | 0.56 | – | – |
| 24–47 | 11 | 0.50 | – | – |
| 48–71 | 16 | 0.61 | – | – |
| 72–95 | 15 | 0.64 | – | – |
| 96–119 | 12 | 0.65 | 5 | 1.58 |
| ≥120 | 14 | 0.65 | 8 | 1.08 |

Adjusted for age (2 year groups), parity (0, 1–2, ≥3 births), age at first term pregnancy (no pregnancy, ≤19, 20–24, ≥25 years) and, for those aged ≥45, age and type of menopause (still menstruating; natural menopause at age <40, 40–44, ≥45; hysterectomy with at least one ovary retained at age <40, 40–44, ≥45; bilateral oophorectomy with or without hysterectomy at age <40, 40–44, ≥45). Age at first term pregnancy was unknown in two clinics, so age at marriage plus one year was substituted. Ages 25–44 years, $\chi_6^2=0.69$ (n.s.). Ages ≥45 years, $\chi_2^2=4.11$ (n.s.).

**Table II** Breast cancer incidence by interval since first oral contraceptive use and age

| *Interval since first oral contraceptive use (months)* | *Ages 25–44* | | *Ages ≥45* | |
|---|---|---|---|---|
| | *Number of cases* | *Rate per 1,000 woman–years* | *Number of cases* | *Rate per 1,000 woman–years* |
| Never used | 49 | 0.62 | 50 | 2.24 |
| ≤47 | 7 | 0.71 | – | – |
| 48–95 | 13 | 0.52 | – | – |
| 96–143 | 20 | 0.57 | – | – |
| 144–191 | 22 | 0.63 | 7 | 2.71 |
| ≥192 | 15 | 0.66 | 6 | 0.80 |

Adjustments as for Table I. Ages 25–44 years, $\chi_5^2=0.71$ (n.s.). Ages ≥45 years, $\chi_2^2=7.87$ ($P<0.05$).

**Table III** Breast cancer incidence by interval since last oral contraceptive use and age

| Interval since last oral contraceptive use (months) | Ages 25–44 | | Ages ≥45 | |
|---|---|---|---|---|
| | Number of cases | Rate per 1,000 woman–years | Number of cases | Rate per 1,000 woman–years |
| Never used | 49 | 0.62 | 50 | 2.24 |
| Current user | 24 | 0.67 | | |
| ≤23 | 17 | 0.86 | 7 | 1.80 |
| 24–47 | 9 | 0.50 | | |
| 48–71 | 6 | 0.35 | | |
| 72–95 | 10 | 0.68 | 6 | 0.90 |
| 96–119 | 4 | 0.39 | | |
| ≥120 | 7 | 0.61 | | |

Adjustments as for Table I. Ages 25–44 years, $\chi^2_7 = 5.4$ (n.s.). Ages ≥45 years, $\chi^2_2 = 4.8$ (n.s.).

**Table IV** Breast cancer incidence by use of oral contraceptives before first term pregnancy and use of oral contraceptives before age 25 years (women aged up to 44 years only)

| Total duration of oral contraceptive use (months) | Use before first term pregnancy | | Use before age 25 years | |
|---|---|---|---|---|
| | Number of cases | Rate per 1,000 woman–years | Number of cases | Rate per 1,000 woman–years |
| Never used | 84 | 0.57 | 108 | 0.62 |
| ≤47 | 15 | 0.83 | 17 | 0.57 |
| ≥48 | 7 | 0.62 | 1 | 0.75 |

Adjustments as for Table I. The data for the two clinics for which age at first term pregnancy was unknown have been omitted from the left hand part of the table, reducing the number of cancers from 126 to 106. Use before first term pregnancy, $\chi^2_2 = 1.80$ (n.s.). Use before age 25 years, $\chi^2_2 = 0.12$ (n.s.).

**Table V** Types of oral contraceptive used at any time by 189 women with breast cancer and 378 matched controls and before first term pregnancy by 103 women up to 44 years of age with breast cancer and 206 matched controls[a]

| OCs used | | OC use at any time | | | | OC use before first term pregnancy | | | |
|---|---|---|---|---|---|---|---|---|---|
| | | Cases | | Controls | | Cases | | Controls | |
| | | No. of users | Total months used | No. of users | Total months used | No. of users | Total months used | No. of users | Total months used |
| *Oestrogen type and dose in pill* | | | | | | | | | |
| Ethinyl-oestradiol | 100 μg | 6 | 56 | 14 | 187 | 1 | 15 | 1 | 17 |
| | 50 μg | 70 | 3,828 | 155 | 8,872 | 16 | 586 | 29 | 991 |
| | 30 μg | 33 | 792 | 64 | 1,528 | 3 | 28 | 3 | 37 |
| | Any dose | 81 | 4,762 | 177 | 10,659 | 17 | 629 | 29 | 1,045 |
| Mestranol | 100 μg | 21 | 625 | 42 | 1,524 | 4 | 96 | 2 | 44 |
| | 50 μg | 33 | 1,707 | 76 | 3,351 | 6 | 126 | 15 | 263 |
| | Any dose | 55 | 2,902 | 128 | 6,092 | 11 | 263 | 21 | 411 |
| *Progestogen type in pill* | | | | | | | | | |
| Norethisterone acetate | | 42 | 2,125 | 102 | 3,968 | 10 | 306 | 14 | 395 |
| Norethisterone | | 42 | 2,182 | 96 | 4,528 | 6 | 129 | 15 | 266 |
| Lynoestrenol | | 28 | 1,078 | 67 | 2,948 | 5 | 182 | 17 | 478 |
| Megestrol acetate | | 14 | 454 | 38 | 1,310 | 2 | 57 | 10 | 171 |
| Ethynodiol diacetate | | 22 | 912 | 47 | 2,744 | 6 | 154 | 4 | 85 |
| Norgestrel/Levonorgestrel | | 40 | 1,044 | 73 | 1,945 | 4 | 67 | 3 | 56 |
| *Major individual pills*[b] | | | | | | | | | |
| Anovlar | | 8 | 254 | 8 | 249 | 1 | 22 | 1 | 4 |
| Gynovlar | | 19 | 765 | 52 | 1,892 | 3 | 82 | 8 | 299 |
| Lyndiol 2.5 | | 14 | 279 | 31 | 681 | 3 | 41 | 8 | 101 |
| Minovlar | | 22 | 814 | 53 | 1,412 | 7 | 202 | 6 | 74 |
| Norinyl-1 | | 27 | 1,300 | 53 | 2,406 | 5 | 120 | 11 | 212 |
| Norlestrin | | 4 | 290 | 12 | 394 | 0 | 0 | 2 | 18 |
| Ovulen | | 14 | 391 | 34 | 1,176 | 4 | 96 | 2 | 39 |
| Ovulen 50 | | 11 | 344 | 27 | 1,334 | 3 | 58 | 2 | 37 |
| Volidan | | 10 | 398 | 29 | 1,082 | 2 | 42 | 9 | 154 |
| Orthonovin 1/50 | | 12 | 407 | 33 | 945 | 1 | 6 | 4 | 51 |
| Minilyn | | 23 | 759 | 53 | 2,165 | 4 | 141 | 15 | 373 |
| Eugynon 30 | | 16 | 386 | 37 | 744 | 0 | 0 | 0 | 0 |

[a]Omitting data for two clinics at which age at first term birth unknown and three cases who could not be matched; [b]For details of steroidal content of the major pills, see Appendix.

**Table VI** Relative risks (95% confidence intervals) of breast cancer associated with oral contraceptive use before first term pregnancy after excluding all such use within the stated period before diagnosis (or equivalent date for the controls)

| *Exclusion period (years)* | *Months use before first term pregnancy* | *All oral contraceptives* | *Oral contraceptives containing ethinyl-oestradiol* | *Oral contraceptives containing mestranol* |
|---|---|---|---|---|
| 0 | Never | 1.00 | 1.00 | 1.00 |
| | 1–12 | 0.48 (0.05– 4.21) | 0.87 (0.15– 4.94) | 0.65 (0.17– 2.45) |
| | 13–47 | 1.66 (0.44– 6.26) | 1.21 (0.28– 5.28) | 0.98 (0.31– 3.07) |
| | ⩾48 | 1.18 (0.28– 4.93) | 1.03 (0.23– 4.53) | 1.61 (0.09–29.07) |
| 6 | Never | 1.00 | 1.00 | 1.00 |
| | 1–12 | 0.46 (0.09– 2.50) | 1.10 (0.20– 5.93) | 0.09 (0.17– 2.30) |
| | 13–47 | 1.40 (0.33– 5.92) | 0.83 (0.14– 4.95) | 1.54 (0.41– 5.77) |
| | ⩾48 | 1.03 (0.23– 4.64) | 0.63 (0.13– 3.05) | 1.60 (0.09–28.90) |
| 10 | Never | 1.00 | 1.00 | 1.00 |
| | 1–12 | 0.72 (0.07– 7.04) | 0.50 (0.05– 5.06) | 0.53 (0.12– 2.42) |
| | 13–47 | 2.44 (0.59–10.00) | 2.36 (0.35–16.11) | 1.50 (0.40– 5.60) |
| | ⩾48 | 0.87 (0.19– 3.97) | 0.60 (0.10– 3.43) | 1.58 (0.09–28.20) |

*Case–control analysis*

For this part of the analysis, we had two sets of data. One concerned all 189 women with breast cancer and 378 controls matched for age, clinic and date of recruitment. The second concerned 103 of the 126 women with breast cancer aged up to 44 years and 206 controls matched for age, date of recruitment and age at first pregnancy. We first confirmed the general findings in the cohort analyses already described above. We then turned our attention to a detailed assessment of possible variation in the effects of different types of pill. Table V illustrates the main results. Data are given separately for lifetime use of oral contraceptives (based on all 189 cases) and for use before first term pregnancy (based on 103 cases). The figures shown are obviously difficult to assess. However, in the absence of an effect, the ratio of months used in the cases to months used in the controls in any category would be expected to be 0.5. If, entirely arbitrarily, we regard preparations showing a ratio greater than 0.7 or less than 0.3 as 'outliers', then the only ones to fall in this category in the overall analysis are Anovlar (ratio 1.02) and Ovulen 50 (ratio 0.26). The data for oral contraceptive use before first term pregnancy are even more difficult to assess because there is little such exposure. However, if we apply the same test criteria as before and, in addition, require there to be at least 100 woman-years of exposure in the controls, then the 'outliers' are norethisterone acetate (ratio 0.78), Gynovlar (ratio 0.27) and Volidan (ratio 0.27). All.in all, although there obviously is variation in exposure to different steroids and individual pills among the cases and controls, we have been unable to discern any clear patterns, either in the data shown in Table V or in other analyses not reproduced here.

Finally, we used the second set of case–control data to investigate a possible latent effect of oral contraceptive use before first term pregnancy. To do this, we excluded successively oral contraceptive use before first term pregnancy within 2, 4,..., 10 years of diagnosis (or the equivalent date for the controls). Estimated relative risks of breast cancer associated with particular durations of oral contraceptive use before first term pregnancy would be expected to change in such an analysis if there was a delayed effect of exposure. The analysis, in fact, showed no trends in relative risk estimate; this was true both for the overall data and for analyses categorised by the type of oestrogen contained in the oral contraceptives used. The data were, however, extremely sparse. Representative findings are given in Table VI.

### Discussion

The findings in studies of oral contraceptive use and breast cancer have recently been reviewed in detail elsewhere (McPherson *et al.*, 1987; Vessey, 1987) and the interested reader is referred to these reviews. In brief, as we stated earlier, there is a consensus that the use of oral contraceptives by women in the middle of the fertile years (say between 25 and 39 years) has no adverse effect on breast cancer risk and our findings add weight to this conclusion. There is, on the other hand, continuing anxiety about the effects of oral contraceptive use at an early age, especially before first term pregnancy. Furthermore, McPherson *et al.* (1987) suggested that any adverse effect might particularly be associated with oral contraceptives containing ethinyl-oestradiol (rather than mestranol) and might be enhanced with the passage of time from exposure. Schlesselman *et al.* (1987, 1988) found no evidence to support these suggestions in the Cancer & Steroid Hormones Study, and likewise, the findings we have presented here are essentially negative. It should be stressed, however, that our data are extremely sparse and that virtually no exposure occurred in the cohort at ages younger than 20 years. Research must continue into the important question of the possible relationship between oral contraceptive use and breast cancer, especially early use.

We thank Mrs C. Brice, Mrs P. Brown, Mrs D. Collinge and the research assistants, doctors, nurses and administrative staff working at the participating clinics for their hard work and loyal support. We are also grateful to the Medical Research Council for financial assistance.

### Appendix

*Oestrogen and progestogen content of oral contraceptives referred to in text and Table V*

| | *Oestrogen (μg)* | *Progestogen (mg)* |
|---|---|---|
| Anovlar | 50 EO | 4.0 NA |
| Gynovlar | 50 EO | 3.0 NA |
| Lyndiol 2.5 | 75 M | 2.5 LE |
| Minovlar | 50 EO | 1.0 NA |
| Norinyl-1 | 50 M | 1.0 N |
| Norlestrin | 50 EO | 2.5 NA |
| Ovulen | 100 M | 1.0 EDD |
| Ovulen 50 | 50 EO | 1.0 EDD |
| Volidan | 50 EO | 4.0 MA |
| Orthonovin 1/50 | 50 M | 1.0 N |
| Minilyn | 50 EO | 2.5 LE |
| Eugynon 30 | 30 EO | 0.25 LN |

Oestrogens: EO, Ethinyloestradiol; M, Mestranol.
Progestogens: NA, Norethisterone acetate; LE, Lynoestrenol; N, Norethisterone; EDD, Ethynodiol diacetate; MA, Megestrol acetate; LN, Levonorgestrel.

### References

McPHERSON, K., VESSEY, M.P., NEIL, A., DOLL, R., JONES, L. & ROBERTS, M. (1987). Early oral contraceptive use and breast cancer: results of another case-control study. *Br. J. Cancer*, **56**, 653.

MEIRIK, O., LUND, E., ADAMI, H.-O., BERGSTROM, R., CHRISTOFFERSEN, T. & BERGSJO, P. (1986). Oral contraceptive use and breast cancer in young women. A joint national case-control study in Sweden and Norway. *Lancet*, **ii**, 650.

PAUL, C., SKEGG, D.C.G., SPEARS, G.F.S. & KALDON, J.M. (1986). Oral contraceptives and breast cancer: a national study. *Br. Med. J.*, **293**, 723.

PIKE, M.C., HENDERSON, B.E., KRAILO, M.D., DUKE, A. & ROY, S. (1983). Breast cancer in young women and use of oral contraceptives: possible modifying effect of formulation and age at use. *Lancet*, **ii**, 926.

SCHLESSELMAN, J.J., STADEL, B.V., MURRAY, P. & LAI, S. (1987). Breast cancer in relation to type of estrogen contained in oral contraceptives. *Contraception*, **36**, 595.

SCHLESSELMAN, J.J., STADEL, B.V., MURRAY, P. & LAI, S. (1988). Breast cancer in relation to early use of oral contraceptives: no evidence of a latent effect. *J. Am. Med. Assoc.*, **259**, 1828.

STADEL, B.V., RUBIN, G.L., WEBSTER, L.A., SCHLESSELMAN, J.J. & WINGO, P.A. (1985). Oral contraceptives and breast cancer in young women. *Lancet*, **ii**, 970.

STADEL, B.V. & SCHLESSELMAN, J.J. (1986). Oral contraceptive use and the risk of breast cancer in women with a 'prior' history of benign breast disease. *Am. J. Epidemiol.*, **123**, 373.

VESSEY, M.P. (1987). Oral contraceptives and breast cancer. *IPPF Med. Bull.*, **21**, no. 6, 1.

VESSEY, M.P., DOLL, R., PETO, R., JOHNSON, B. & WIGGINS, P. (1976). A long-term follow-up study of women using different methods of contraception – an interim report. *J. Biosoc. Sci.*, **8**, 373.

VESSEY, M.P., McPHERSON, K. & DOLL, R. (1981). Breast cancer and oral contraceptives: findings in Oxford–Family Planning Association contraceptive study. *Br. Med. J.*, **282**, 2093.

VESSEY, M.P., McPHERSON, K., YEATES, D. & DOLL, R. (1982). Oral contraceptive use and abortion before first term pregnancy in relation to breast cancer risk. *Br. J. Cancer*, **45**, 327.

VILLARD-MACKINTOSH, L., COLEMAN, M.P. & VESSEY, M.P. (1988). The completeness of cancer registration in England: an assessment from the Oxford–FPA contraceptive study. *Br. J. Cancer*, **58**, 507.